# Supplementary material for: Cyclin B Export to the Cytoplasm via the Nup62 Subcomplex and Subsequent Rapid Nuclear Import Are Required for the Initiation of Drosophila Male Meiosis
Source: Cells. 2023 Nov 11;12(22):2611. doi: 10.3390/cells12222611 (PMC10670764; doi:10.3390/cells12222611)
Supplement: Supplementary file 1 [file cells-12-02611-s001.zip › cells-2684197-supplementary.pdf]

Supplementary figure legends

Fig. S1. Effect of ectopic expression of normal CycB or NLS-CycB on their subcellular localization in mature spermatocytes.

(a-p) Anti-CycB immunostaining of spermatocytes at S5 (a, d, f, i, l, o), S6 (b, e, g, j, m, p), and Prophase I (Pro) (c, h, k, n). The immunofluorescence was observed in normal spermatocytes (a-c), the cells expressing normal CycB (f-h) or NLS-CycB (l-n), *Nup62*-silenced spermatocytes (d, e), the silenced cells expressing normal CycB (i-k) or NLS-CycB (o, p). Spermatocytes expressing normal CycB or NLS-CycB, both of which are fused with HA tag, were simultaneously immunostained with anti-HA antibody. Anti-CycB immunofluorescence (red in a-p, white in a'-p'), Sa-GFP fluorescence to determine the stages of growth phase (green in a-e, white in a''-e'' and f''''-p''''), DNA staining with DAPI (blue in a-p, white in a'''-p'''), anti-HA immunofluorescence (green in f-p, white in f''-p''), and phase-contrast images (f'''''-p'''''). Scale bar: 10μm.

Fig. S2. Control experiment for the In Situ Proximity ligation assay (PLA) in spermatocytes.

(a, b) A negative control experiment with anti-Cdk1 antibody alone in normal spermatocytes at the S5 (a) and S6 (b) phases. 2.1% of spermatocytes showed PLA foci (1/48 cells). (c, d) A negative control experiment with anti-Cdk7 antibody alone in normal spermatocytes at the S5 (c) and S6 (d) phases. 4.7% of spermatocytes showed PLA foci (5/107). (e, f) A negative control experiment with anti-GFP antibody alone in spermatocytes expressing Twine-GFP at the S5 (e) and S6 (f) phases. 3.3 % of spermatocytes showed PLA foci (5/150 cells). (g, h) A positive control experiment with anti-Cdk1 and anti-CycB antibodies in normal spermatocytes at the S5 (g) and S6 (h) phases. 98.2% of spermatocytes showed PLA foci (108/110 cells). Scale bar: 10μm.

Fig. S3. Intracellular localization of Wee1 and Myt1, and their close association with Cdk1 by

the PLA in mature spermatocytes at S5, S6, and ProI.

(a-f) Fluorescent images of spermatocytes expressing Wee1-GFP (a-c) or Myt1-GFP (d-f) at S5 (a, d), S6 (b, e), and Prophase I (Pro) (c, f) stages. Wee1-GFP fluorescence (green in a-c, white in a'-c'), Myt1-GFP fluorescence (green in d-f, white in d'-f'), DNA staining with DAPI (magenta in a-f white in a''-f''). Scale bar: 10 μm. (g-j) Detection of protein complexes containing Cdk1 and Wee1-GFP or Myt1-GFP by the PLA in the spermatocytes expressing Wee1-GFP (g, h) or Myt1-GFP (i, j) at premeiotic stages using ant-Cdk1 and anti-GFP antibodies. Scale bar: 10 μm. 98.8% of spermatocytes expressing Wee1-GFP showed the PLA foci in the whole region (162/164). All spermatocytes expressing Myt1-GFP showed the PLA foci in the whole region (n = 214).

Fig. S4. The effect of *polo*-silencing in spermatocytes on the G2/M progression in male meiosis.

(a) A single intact cyst of spermatids in the testis harboring the *polo*-silenced spermatocytes (*bam*> *poloRNAi*). A phase-contrast image of spermatids stained with DAPI (red). The spermatid cyst consists of 16 cells (10 cysts / 10 cysts examined). Each spermatid contains a Nebenkern and 4 small nuclei (90.8% of 142 cells examined). (b-d) Anti-MPM2 immunostaining of *polo*-silenced spermatocytes at S5 (b), S6 (c), and ProI (d). Anti-MPM2 immunostaining (red in b-d, white in b'-d'), Sa-GFP fluorescence (green in b-d, white in b''-d''), and DNA staining with DAPI (blue in b-d, white in b'''-d'''). Scale bar: 10μm.

Fig. S5. Subcellular localization of Polo-GFP in spermatocytes from S5 of the growth phase to the later ProI.

(a-h) Anti-CycB immunostaining of spermatocytes expressing GFP-tagged Polo at S5 (a, g), S6 (b, h), and ProI (c-f) with the progression of meiosis. The immunofluorescence was observed in normal spermatocytes (*bam>Polo-GFP*) (a-f) and *Nup62*-silenced spermatocytes (*bam>Nup62RNAi, Polo-*

GFP) (g, h). GFP fluorescence of Polo-GFP (green in a-h, white in a’-h’), anti-CycB immunostaining (red in a-h, white in a”-h”), and DNA staining with DAPI (blue in a-h, white in a”’-h”’). Scale bar: 10μm.

Fig. S6. Examination of protein interaction between CycB and Polo-GFP by *in situ* proximity ligation assay (PLA).

(a-e) *In situ* PLA to detect the protein complexes containing CycB and Polo-GFP in the spermatocytes expressing Polo-GFP at S5 (a, d), S6 (b, e), and ProI (c) using anti-CycB and GFP antibodies. Normal spermatocytes (*bam>Polo-GFP*) (a-c) and *Nup62*-depleted spermatocytes (*bam>Nup62RNAi, Polo-GFP*) (d, e). Scale bar: 10μm. (*bam>Polo-GFP*: n = 343, *bam>Nup62RNAi, Polo-GFP*: n = 273).

Fig. S7. Intracellular localization of Z600 protein in normal and *Nup62*-silenced spermatocytes at mature stages, and immunostaining to detect colocalization of Z600 and CycB in the premeiotic cells.

(a-f) Immunostaining of mature spermatocytes with anti-Z600 antibody. Normal control (a-c), and *Z600*-silenced (d, e) or *Nup62*-silenced (f) spermatocytes. Immunofluorescence of the cells with anti-Z600 antibody (red in a-f, white in a’-f’), Sa-GFP fluorescence (green in a-e, white in a”-e”), DNA staining with DAPI (blue in a-f, white in a”’-e”’, f”), and phase-contrast images (a””-e””, f”’). (d, e) Anti-Z600 immunostaining of the *Z600*-silenced cells at S5 (d) and S6 (e) shows a faint signal at the background level. Scale bar: 10μm. (g, h) Simultaneous immunostaining of premeiotic spermatocytes with anti-HA and anti-CycB antibodies at S5 (g) and S6 (h) stages. Anti-HA immunostaining (red in g, h, white in g’, h’), anti-CycB immunostaining (green in g, h, white in g”, h”), DNA staining with DAPI (blue in g, h, white in g”’, h”’), Sa-GFP fluorescence (white in g””, h””), and phase-contrast images (g””’, h””’). Scale bar: 10μm.

Fig. S8. Single intact cysts of spermatids at onion stage in the testes harboring spermatocyte-specific silencing of components required for the nucleo-cytoplasmic transport.

(a-d) Phase-contrast images of intact cysts consisting of spermatids harboring round Nebenkerns from control testis (a) or testes with spermatocytes-specific silencing of *Nup62* (b), *emb* (c), or *Fs(2)Ket* (d). DNA staining with DAPI (red). Scale bar: 10µm. No spermatid cysts containing 64 cells were found in testes harboring the *Nup62*- (n = 8), *emb*- (n = 20), *Fs(2)Ket*-silenced spermatocytes (n = 12).

Yamazoe., et al

Figure. S1

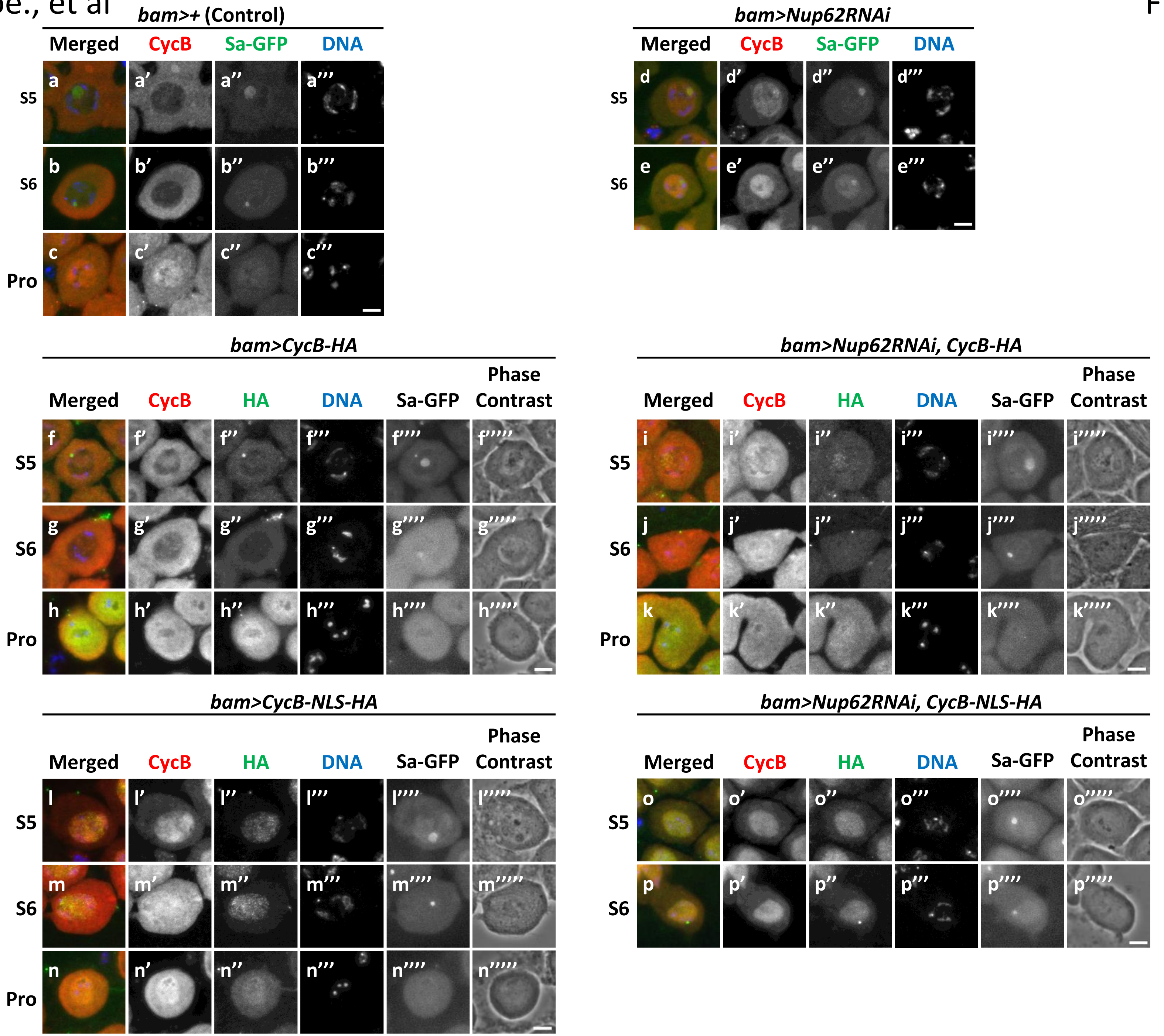

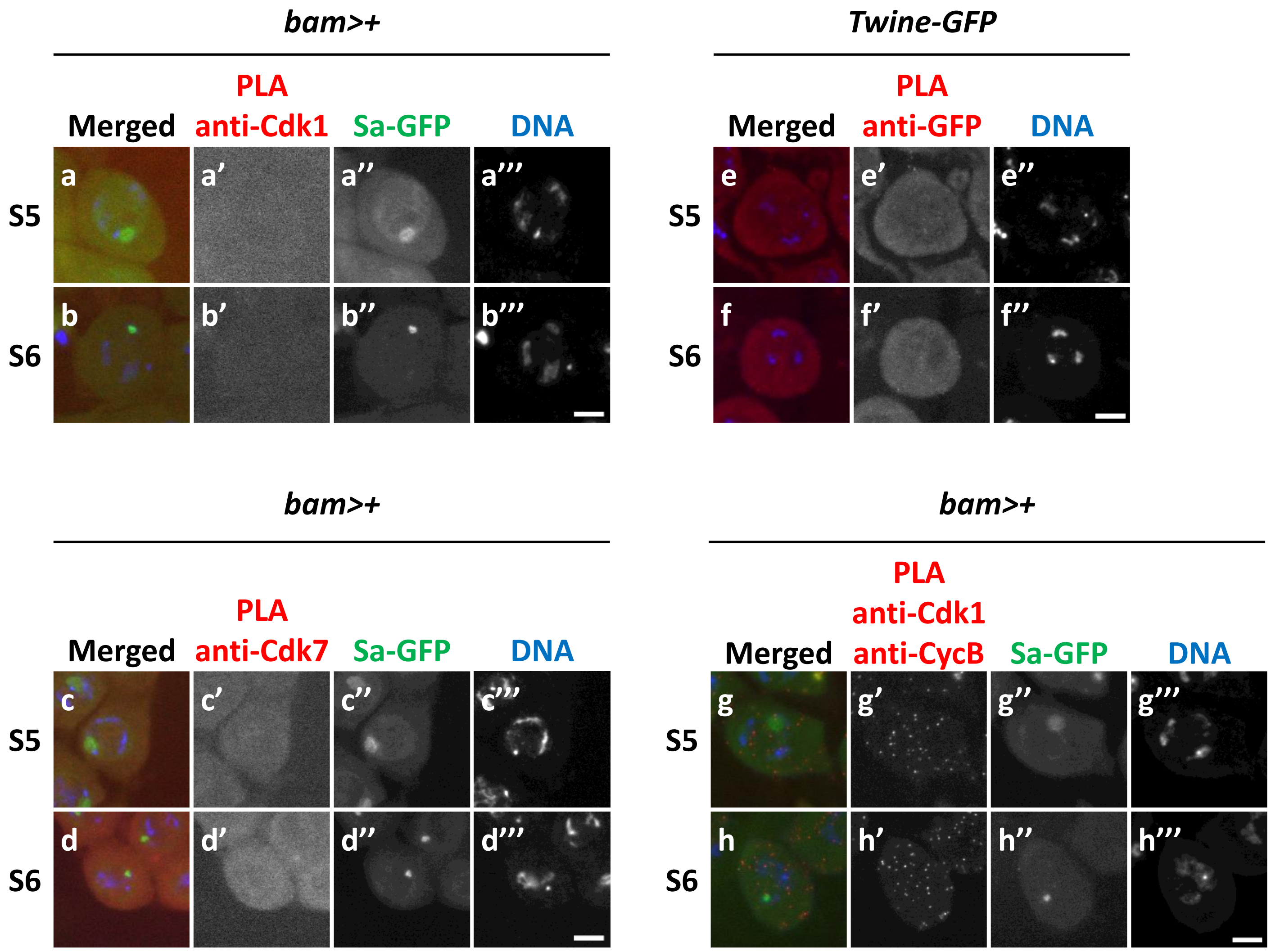
bam>+
PLA
Merged
anti-Cdk1
Sa-GFP
DNA
S5
S6
a a' a'' a'''
b b' b'' b'''
Twine-GFP
PLA
Merged
anti-GFP
DNA
S5
S6
e e' e''
f f' f''
bam>+
PLA
Merged
anti-Cdk7
Sa-GFP
DNA
S5
S6
c c' c'' c'''
d d' d'' d'''
bam>+
PLA
anti-Cdk1
anti-CycB
Merged
Sa-GFP
DNA
S5
S6
g g' g'' g'''
h h' h'' h'''

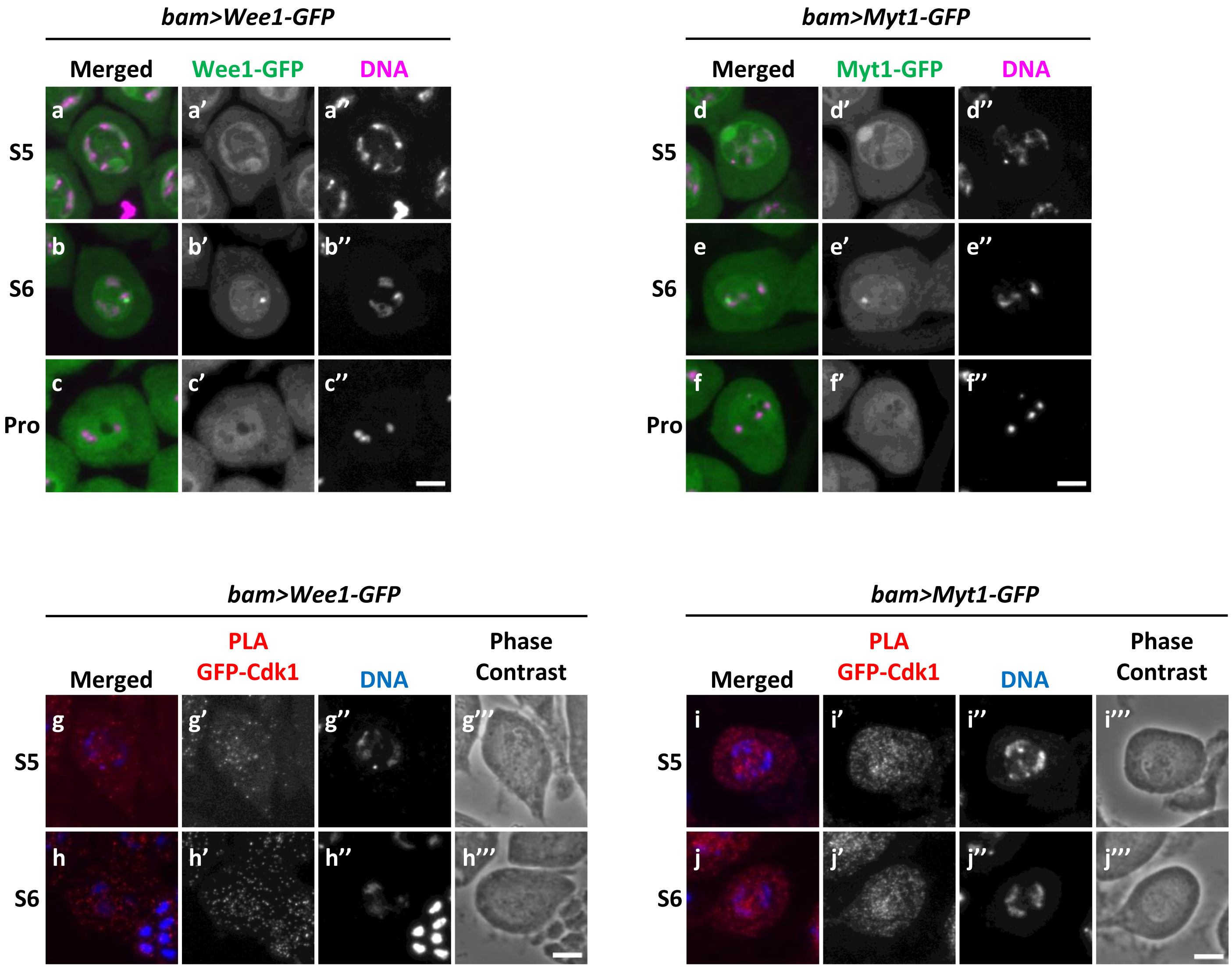
bam>Wee1-GFP
Merged
Wee1-GFP
DNA
S5
S6
Pro
a
a'
a''
b
b'
b''
c
c'
c''
bam>Myt1-GFP
Merged
Myt1-GFP
DNA
S5
S6
Pro
d
d'
d''
e
e'
e''
f
f'
f''
bam>Wee1-GFP
Merged
PLA
GFP-Cdk1
DNA
Phase
Contrast
S5
S6
g
g'
g''
g'''
h
h'
h''
h'''
bam>Myt1-GFP
Merged
PLA
GFP-Cdk1
DNA
Phase
Contrast
S5
S6
i
i'
i''
i'''
j
j'
j''
j'''

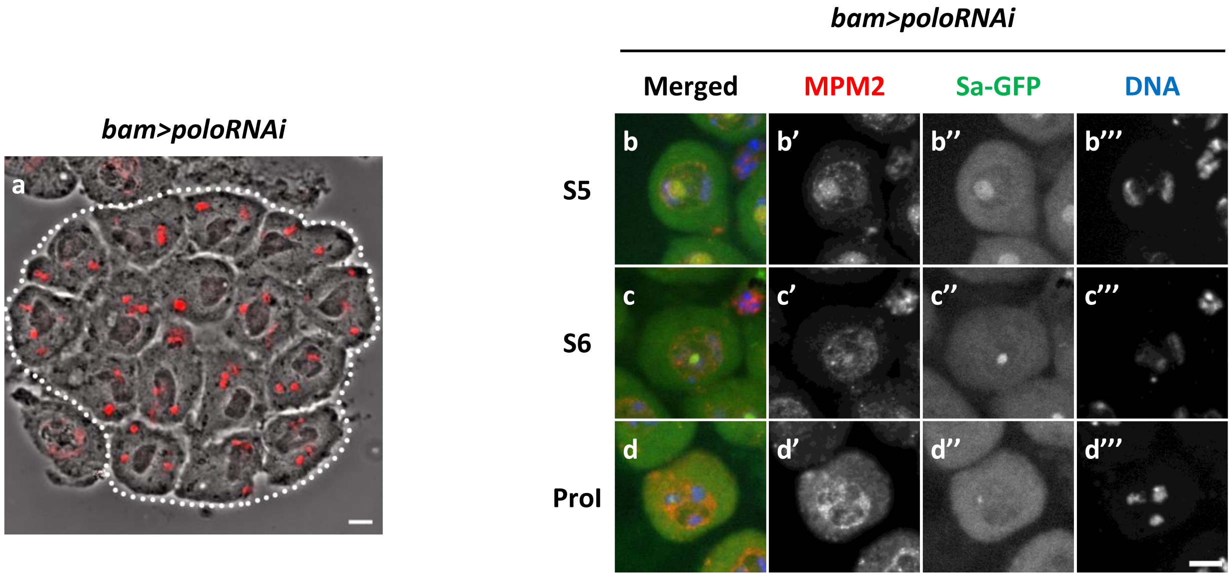
bam>poloRNAi
a
bam>poloRNAi
Merged
MPM2
Sa-GFP
DNA
S5
S6
Prol
b
b’
b’’
b’’’
c
c’
c’’
c’’’
d
d’
d’’
d’’’

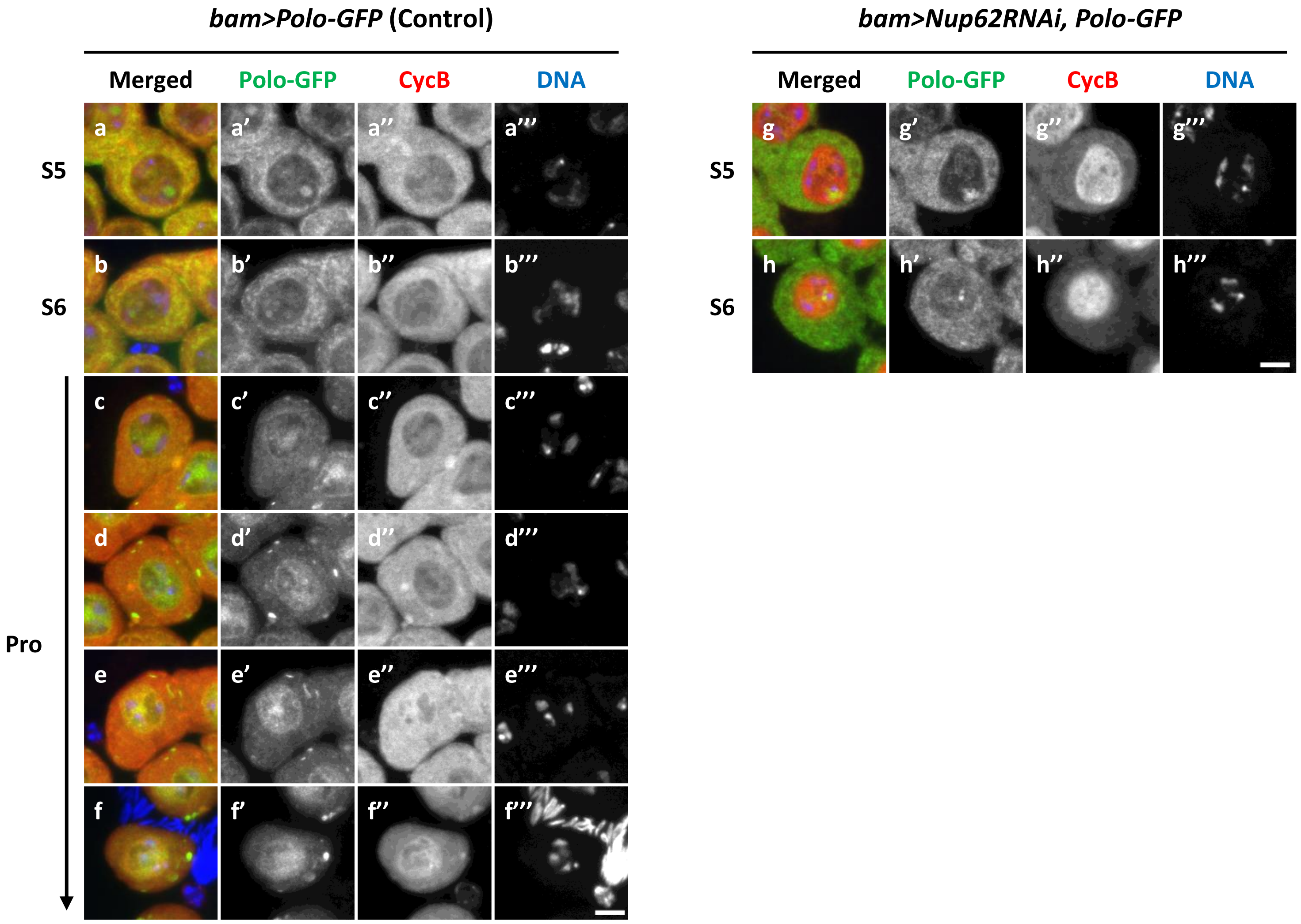
bam>Polo-GFP (Control)
bam>Nup62RNAi, Polo-GFP
Merged
Polo-GFP
CycB
DNA
S5
S6
Pro
a a’ a’’ a’’’
b b’ b’’ b’’’
c c’ c’’ c’’’
d d’ d’’ d’’’
e e’ e’’ e’’’
f f’ f’’ f’’’
g g’ g’’ g’’’
h h’ h’’ h’’’

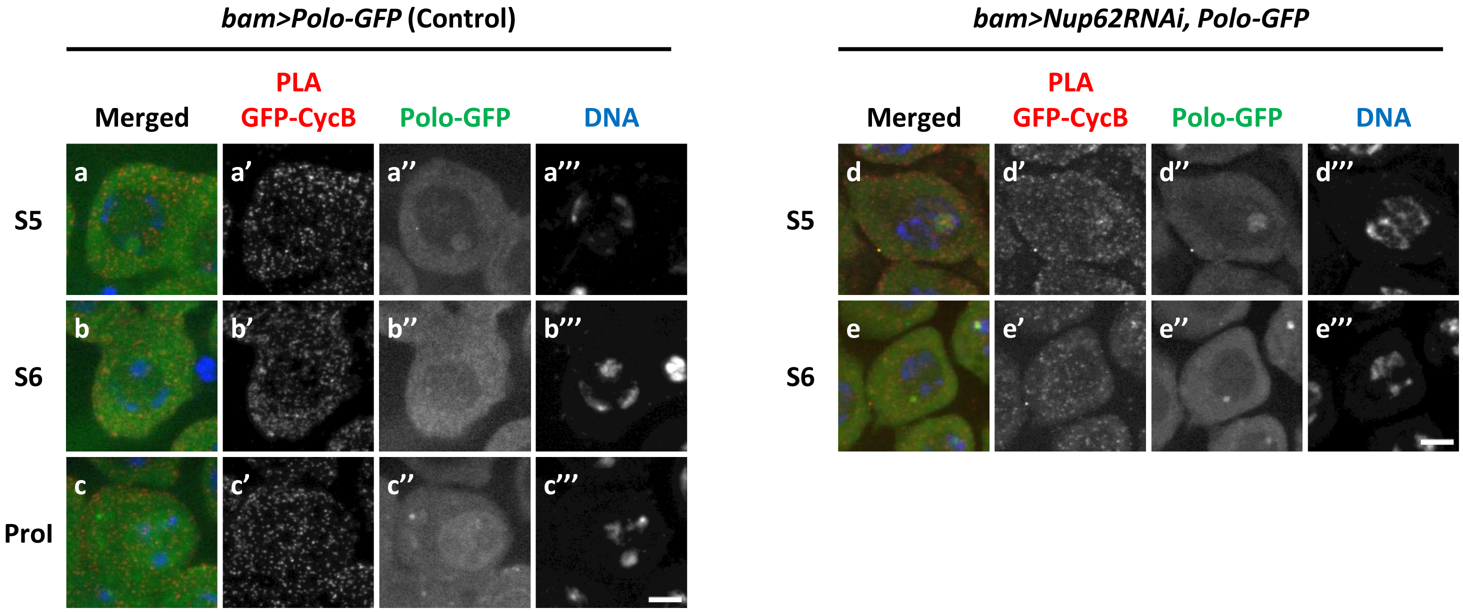

bam>Polo-GFP (Control)
Merged
PLA
GFP-CycB
Polo-GFP
DNA
S5
S6
ProI
a
a’
a’’
a’’’
b
b’
b’’
b’’’
c
c’
c’’
c’’’
bam>Nup62RNAi, Polo-GFP
d
d’
d’’
d’’’
e
e’
e’’
e’’’

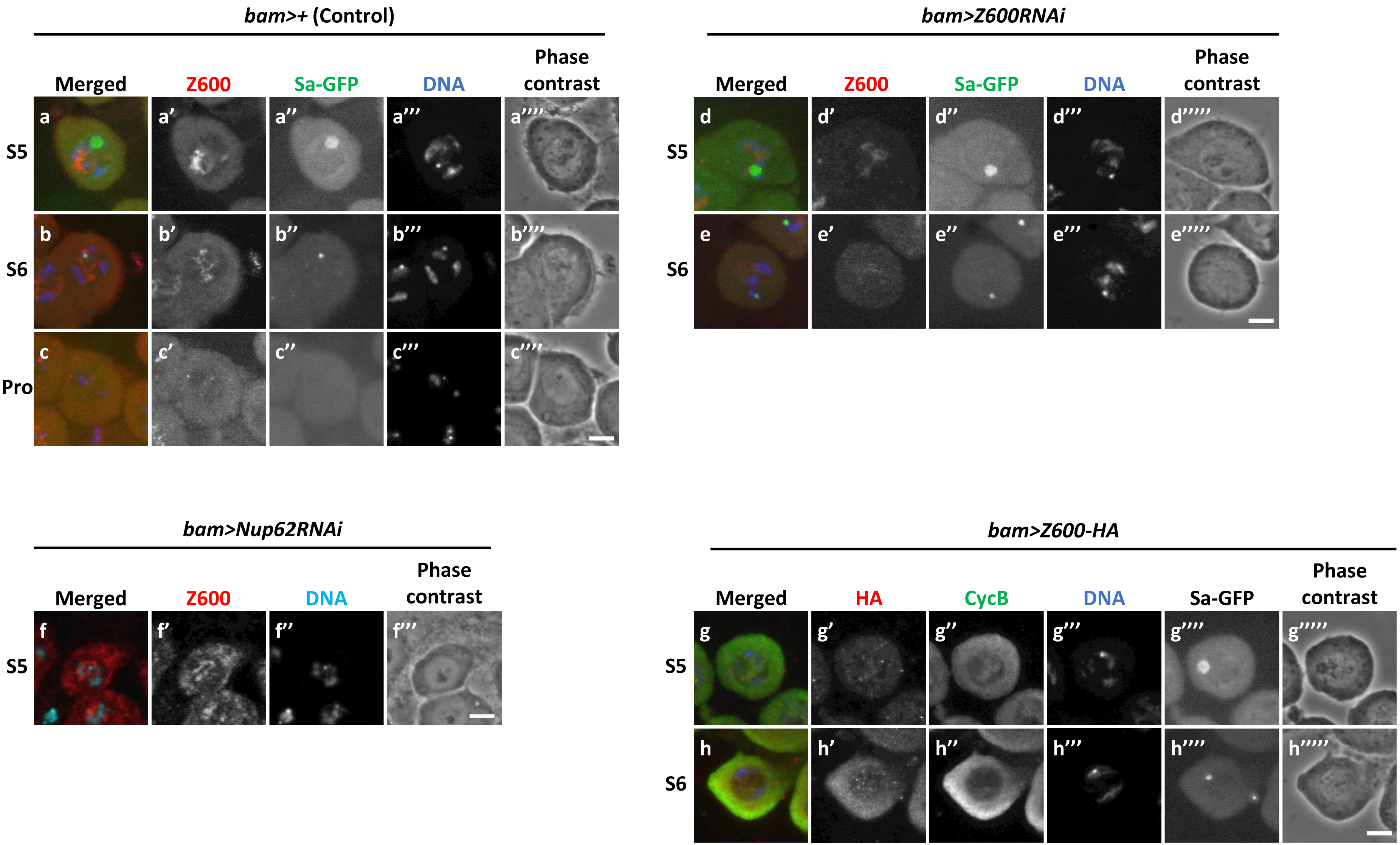
bam>+ (Control)
Merged
Z600
Sa-GFP
DNA
Phase contrast
S5
S6
Pro
bam>Z600RNAi
Merged
Z600
Sa-GFP
DNA
Phase contrast
S5
S6
bam>Nup62RNAi
Merged
Z600
DNA
Phase contrast
S5
bam>Z600-HA
Merged
HA
CycB
DNA
Sa-GFP
Phase contrast
S5
S6

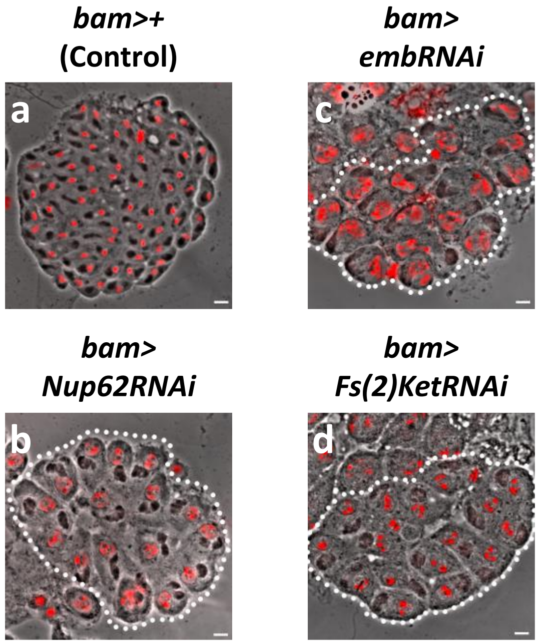
bam>+
(Control)
bam>
embRNAi
a
c
bam>
Nup62RNAi
bam>
Fs(2)KetRNAi
b
d
